# A New Forecasting Approach for Oil Price Using the Recursive Decomposition–Reconstruction–Ensemble Method with Complexity Traits

**DOI:** 10.3390/e25071051

**Published:** 2023-07-12

**Authors:** Fang Wang, Menggang Li, Ruopeng Wang

**Affiliations:** 1 School of Economics and Management, Beijing Jiaotong University, Beijing 100044, China; 2 Beijing Laboratory of National Economic Security Early-Warning Engineering, Beijing Jiaotong University, Beijing 100044, China; 3 National Academy of Economic Security, Beijing Jiaotong University, Beijing 100044, China; 4 Department of Mathematics, Beijing Institute of Petrochemical Technology, Beijing 100044, China; wangruopeng@bipt.edu.cn

**Keywords:** oil price forecasting, complexity trait, component reconstruction, recursive CEEMDAN algorithm, decomposition–reconstruction–ensemble model

## Abstract

The subject of oil price forecasting has obtained an incredible amount of interest from academics and policymakers in recent years due to the widespread impact that it has on various economic fields and markets. Thus, a novel method based on decomposition–reconstruction–ensemble for crude oil price forecasting is proposed. Based on the Complete Ensemble Empirical Mode Decomposition with Adaptive Noise (CEEMDAN) technique, in this paper we construct a recursive CEEMDAN decomposition–reconstruction–ensemble model considering the complexity traits of crude oil data. In this model, the steps of mode reconstruction, component prediction, and ensemble prediction are driven by complexity traits. For illustration and verification purposes, the West Texas Intermediate (WTI) and Brent crude oil spot prices are used as the sample data. The empirical result demonstrates that the proposed model has better prediction performance than the benchmark models. Thus, the proposed recursive CEEMDAN decomposition–reconstruction–ensemble model can be an effective tool to forecast oil price in the future.

## 1. Introduction

Crude oil, which is the world’s most important chemical raw material and strategic resource, ensures the normal operation of the national economy and people’s livelihoods, and it is a critical support for the development of the entire modern industrial society. Crude oil plays an important role in the global economy, political situation, and military strength of various countries as a basic energy source. As a result, changes in crude oil prices have sparked widespread concern worldwide. Because of the interactive impact of various factors such as the global economy, exchange rate changes, speculative behavior, and geopolitics, the oil price always exhibits non-linearity, non-stationarity, and high complexity, which poses significant challenges to crude oil price forecasting.

In the literature, various linear and nonlinear models have been used separately or in combination to make forecast (see, e.g., Buyuksahin & Ertekin [1]). Linear methods assume that a given time series is regular with no sudden movements. It becomes challenging because sudden movements with variation and extreme values are normal in many real-world time series such as financial data and renewable energy data (see, e.g., Xu et al. [2]). Numerous nonlinear time series prediction methods (see, e.g., Kantz & Schreiber [3]) have been proposed in the literature to capture these nonlinearities. Conventional linear methods can better approximate time series with no high volatility and multicollinearity. Zhang et al. [4] and Elman [5] show that nonlinear methods have the advantages when modeling a complex structure in time series with high accuracy. No universal model is suitable for all circumstances because each type of method outperforms others in different domains. Individually capturing general patterns in the time series data using only one linear or nonlinear model appears to be difficult (see, e.g., Khashei & Bijari [6]). To overcome this limitation, Taskaya & Casey [7] proposed hybrid techniques with both linear and nonlinear models. The hybrid methodology is a synthesis of various prediction methods. It is usually a combination of traditional econometric models and AI algorithms (see, e.g., Wang et al. [8]) or a combination of different econometric models or AI algorithms.

In addition to the hybrid methodology, the ensemble learning algorithm is an important paradigm to overcome the limitations of single methods. Both hybrid methodology and the ensemble method consider the shortcomings of single models. With the divide-and-conquer strategy (see, e.g., Yu et al. [9] and Dong et al. [10]), the decomposition–ensemble learning methods are an important branch of ensemble learning paradigms. Because it will take a lot of time to make individual prediction from all decomposed components, the number of decomposed components is necessarily reduced. Yu et al. [11] first proposed a decomposition–ensemble model with a reconstruction step that considered some data characteristics. Recently, Yu & Ma [12] introduced a memory-trait-driven reconstruction method into the decomposition and ensemble framework. Inspired by their work, a new model based on decomposition–ensemble learning with a reconstruction step that considers the data complexity traits is used to explore the price predictions of crude oil. In this model, all steps of mode reconstruction, component prediction, and ensemble prediction are driven by complexity traits. First, a decomposition–ensemble approach is used to decompose the oil price time series. Second, the complexity of these decomposed components are separately computed. Then, each component can be identified based on its complexity ranking from high to low. Different components are predicted through appropriated models. Finally, the forecasting for different components can be aggregated to produce the final prediction output. The contributions of the article are as follows:

- A novel decomposition–reconstruction–ensemble method is proposed with clustering capability to capture the inner complexity traits. The performance of the proposed recursive CEEMDAN for different complexity traits of data is tested and validated using popular single models and several decomposition–reconstruction–ensemble models.
- The proposed recursive CEEMDAN technique is used to improve the performance of the CEEMDAN decomposition method by recursively decomposing the rapidly fluctuating components into less volatile sub-components.
- In the proposed recursive CEEMDAN decomposition–reconstruction–ensemble forecasting methodology, the reconstruction method, prediction method, and ensemble method are determined by the complexity traits of the crude oil data themselves.

The remainder of this paper is organized as follows. Section 2 considers a comparison to the related works. Research data and the decomposition–reconstruction–ensemble method are discussed in Section 3. Section 4 presents the error measures to validate the prediction models. Some main findings are illustrated by comparing the results of the proposed model to the benchmark models. The prediction performance of the proposed model is further discussed in Section 5. Section 6 summarizes this paper and provides the improvement direction of future research.

## 2. Related Work

### 2.1. Forecasting by Statistical Models

Statistical models, which are also known as random time series models, include exponential smoothing (ES) (see, e.g., Kourentzes et al. [13]), auto-regressive integrated moving average (ARIMA) model (see, e.g., Guo [14]), generalized auto-regressive conditional heteroskedasticity (GARCH) model (see, e.g., Zhang et al. [15]), hidden Markov model (HMM) (see, e.g., Isah & Bon [16]), and vectorial auto-regression (VAR) (see, e.g., Mirmirani & Li [17]). For example, Zolfaghari & Gholami [18] showed that ARIMA models had a good forecasting impact on international crude oil prices. To modify the mean and variance of the log returns of crude oil prices, Zhu et al. [19] introduced a hidden Markov model to obtain the behavior of random events and subjective factors for time series fluctuations. Using a VAR model, Drachal [20] applied the global economic policy uncertainty index, production, volatility index, and crude oil volatility to predict crude oil prices. Despite their simplicity and ease of implementation, these statistical models cannot directly process time series with nonlinear characteristics due to their linear correlation structure. Meanwhile, as the soft computing technology has advanced, many different intelligent algorithms have been developed and widely used in various data predictions. However, conventional statistical and econometric models are constrained by stringent theoretical assumptions, including linearity, stationarity, and dependence on specific distributional properties. As a result, these methods may encounter limitations in accurately forecasting wind power time series that are non-stationary, nonlinear, and characterized by complex dynamics.

### 2.2. Forecasting by Artificial Intelligence and Machine Learning Methods

A crucial presumption in the application of econometric models is that the time series data under study are a linear process. However, crude oil prices do not satisfy this requirement, which can result in less accurate forecasting outcomes. In contrast, various nonlinear intelligence and machine learning methods (e.g., the support vector machine (SVM) proposed by Yu et al. [21] and the extreme learning machine (ELM) proposed by Wang et al. [22]) have emerged to satisfy the requirements, and they can be applied to time series prediction tasks. Moreover, deep learning is gaining popularity in machine learning, since conventional machine learning techniques employ shallow structures. Recently, an artificial neural network (ANN) (see, e.g., Jammazi & Aloui [23]), a back-propagation neural network (BPNN) (see, e.g., Khashei & Bijari [6]), long short-term memory (LSTM) networks (see, e.g., Urolagin et al. [24]), and convolutional neural networks (CNNs) (see, e.g., Li et al. [25]) can implement time series with nonlinear characteristics and have high prediction precision. For example, Wang & Wang [26] created a crude oil price forecasting model that utilized a random Elman recurrent neural network, and the predictive power of the model was analyzed in comparison to other models. Yu et al. [27] incorporated the cutting-edge AI method of EELM into an ensemble model formulation to forecast crude oil prices, and findings showed that the suggested unique ensemble learning paradigm statistically outperformed all investigated benchmark models. However, these models have some drawbacks, including local minima, over-fitting, and a large sample size. While it has been demonstrated that ensemble models can outperform individual models, they are still susceptible to issues such as overfitting and being trapped in local extrema, which can limit their ability to generalize effectively.

### 2.3. Forecasting by Hybrid Models

To overcome the limitations of the aforementioned techniques, hybrid models have been proposed. It is not uncommon for researchers to employ a combination of econometric models and artificial intelligence algorithms or even a combination of econometric models and artificial intelligence algorithms. For example, Cheng et al. [28] predicted crude oil prices in 2018 using the vector error correction and nonlinear auto-regressive neural network (VEC-NAR) model. To enhance the technical indicator-based crude oil price forecasting, He et al. [29] implemented a unique hybrid forecast approach using scaled principal component analysis (s-PCA). In-sample and out-of-sample performance comparisons revealed that the s-PCA model was superior to the compared models. Wang & Fang [30] developed a novel combination of the FNN model and stochastic time effective function for crude oil prices forecasting, i.e., the WT-FNN model, and the findings revealed that the WT-FNN model had the best predictive impact. Zhang et al. [15] offered a novel hybrid technique to predict crude oil prices based on the least square support vector machine, particle swarm optimization, and GARCH model. The experimental findings demonstrated that this approach might accurately estimate crude oil prices. To predict crude oil prices accurately, Wang et al. [31] employed a Markov model to implement the GARCH-MIDAS model for both short-term and long-term state conversion, but they discovered that short-term predictions were more accurate. Like the hybrid approach, our proposed decomposition–ensemble method also takes into account the shortcomings of single models. The biggest difference is that the ensemble learning employs several identical individual methods for ensemble prediction.

### 2.4. Forecasting by the Decomposition–Ensemble Learning Method

Recent studies have established a novel ensemble predicting approach called the decomposition ensemble to manage the challenge of forecasting nonlinear time-series data. Similar to the hybrid method, this approach considers the limitations of single models. Ensemble learning employs multiple identical single techniques for ensemble prediction, whereas the hybrid model employs multiple distinct single models for combination prediction. Oil price predictions typically rely on various significant studies. For example, Li et al. [25] and Li et al. [32] decomposed the monthly crude oil futures price data into multiple modes using VMD. Then, they forecast each mode using a SVM that was optimized by a genetic algorithm and a BPNN that was optimized by a genetic algorithm. Using the Akaike information criterion (AIC) to determine a reasonable lag, Ding [33] proposed a decomposition ensemble model using ensemble empirical mode decomposition (EEMD) for crude oil forecasting. Yu et al. [9] used empirical mode decomposition (EMD) to decompose crude oil prices and the feedforward neural network (FNN) to forecast the components. Zheng et al. [34] recently proposed a method combining an empirical mode decomposition algorithm, quadratic surface support vector regression, and the autoregressive integrated moving average method for the stock indices and future price forecasting. The study obtained better forecasting results than the direct forecasting model. However, the existing literature on constructing the decomposition–ensemble framework has some limitations. It primarily focuses on selecting decomposition–reconstruction–prediction–ensemble methods based on the characteristics of the model, rather than taking into account the characteristics of the data themselves. Therefore, the method proposed in this paper has the ability of selecting appropriate decomposition methods, reconstruction methods, prediction methods, and ensemble methods based on the specific traits of the data.

## 3. Methodology

### 3.1. Recursively Decomposition Method

**Figure 1:**
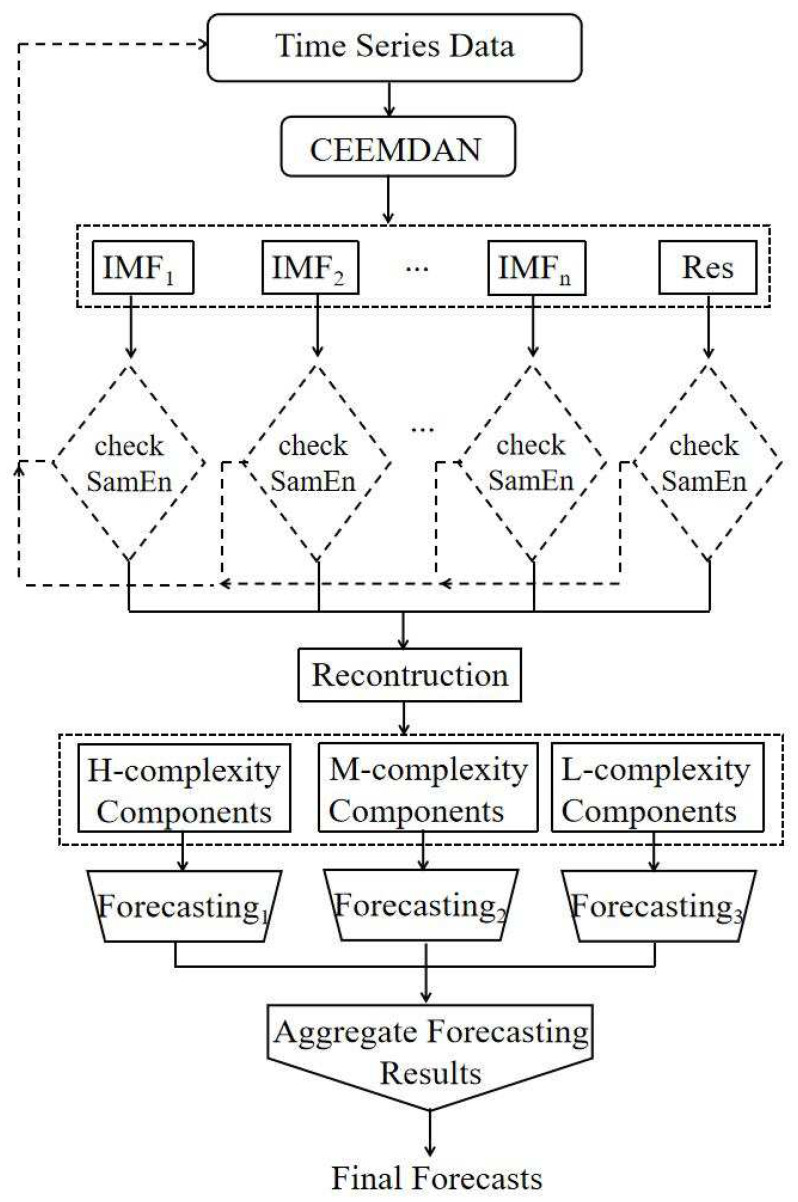
General framework of the recursive CEEMDAN decomposition–reconstruction–ensemble methodology.

In this paper, we propose a recursive CEEMDAN-based technique for time series forecasting, which attempts to extract more stable sub-components from rapidly changing components to improve the prediction accuracy. The architecture of the proposed method is given in Figure 1.

The proposed method recursively calls the CEEMDAN decomposition technique (see, e.g., Torres et al. [35]) for each component until it satisfies one of the following two conditions:

- The component becomes less complex than the given series.
- The correlation between the component and the given series exceeds a specified threshold.

The first condition takes into account the sample entropy values of each component. According to the methods proposed by Richman & Moorman [36], the sample entropy value is greater for more complicated components. Therefore, the more complicated components are decomposed again into their own sub-components via CEEMDAN in the algorithm.

The second condition employs Pearson correlation (see, e.g., Hauke & Kossowski [37]) to determine the similarity between the specified component and the series. High correlation is a termination criterion for this recursive method. Recursive decomposition is halted if a sub-component is substantially connected with its higher component regardless of its fluctuation rate.

The pseudo-code of the proposed method is indicated in Algorithm 1. After the given data have been decomposed into components via CEEMDAN at line 1, all components are called in the loop at line 2 where each component is analyzed. At line 3, a sample entropy value of each component and the input data, which is the first condition of the proposed algorithm, is calculated using the sample entropy (SE) method (see, e.g., Richman & Moorman [36]). At line 4, the correlation results are obtained. At line 5, the second condition of the proposed algorithm which measures the correlation between upper component and the sub-component is calculated. If the sub-component has a higher sample entropy value than its upper component and their correlation is low, the given component must be re-decomposed by CEEMDAN. In this case, the Recursive Method function is called again for this sub-component at line 7. Otherwise, no more decomposition is required for the sub-component. At line 8, the ultimate decomposition result is formed.
**Algorithm 1:** Recursive CEEMDAN-based decomposition methodInput: the original time series dataOutput: the decomposed time series1: Decompose the input time series data with the method CEEMDAN (different IMFs are obtained).2: **for** each component imf in IMFs **do**3: Compute each component’s and the input data’s sample entropy $imf_{SE}$, $inputdata_{SE}$.4: Derive the correlation between the selected component imf and the input data $imf_{corr}$.5: **if** $imf_{SE}$ <$inputdata_{SE}$ and $imf_{corr}$ <0.9**then**6: Output the selected component imf.**else**7: Go to step 1 and decompose the selected imf.8: **return** The decomposed imfs and their sub-components.

Then, based on the recursive CEEMDAN algorithm, different decomposed components of the original data and their sub-components are obtained. The decomposed components are identified as low-complexity components when they have smaller complexity traits than the original time series after the first decomposition. The decomposed components with larger complexity traits than the original time series will be recognized as high-complexity components when they are recursively decomposed only once. Then, other decomposed components are recognized as medium-complexity components, which implies that these components have larger complexity traits than the original time series and they will be recursively decomposed two or more times.

### 3.2. Performance Evaluation Criteria

To verify the validity of a forecast, the model outcomes are assessed. Numerous experiments are conducted to evaluate the forecasting performance of the proposed hybrid model and the reference models. In this paper, we use three popular accuracy measures with the following corresponding definitions:

(1) $$\begin{array}{ccc} \mathrm{MAE} & = & \frac{1}{N}\sum_{t=1}^{N}\left|d_{t}-O_{t}\right|, \end{array}$$

(2) $$\begin{array}{ccc} \mathrm{RMSE} & = & \sqrt{\frac{1}{N}\sum_{t=1}^{N}{\left(d_{t}-O_{t}\right)}^{2}}, \end{array}$$

(3) $$\begin{array}{ccc} \mathrm{MAPE} & = & \frac{1}{N}\sum_{t=1}^{N}\left|\frac{d_{t}-O_{t}}{d_{t}}\right|, \end{array}$$

where $d_{t}$ and $O_{t}$ are the real and predicted values at time *t* (t=1,2,…,N); *N* is the number of samples in the testing data set; and $\hat{d_{t}}$ and $\hat{O_{t}}$ are the average values of the actual value and predicted value, respectively.

In addition, a Diebold–Mariano (DM) test (see, e.g., Yu et al. [38]) is chosen to prove the superiority of the proposed model. Furthermore, popular single models and several decomposition–reconstruction–ensemble models are built as benchmark models to test the effectiveness of the proposed model. In detail, ES is constructed as the single benchmark model for the traditional econometric model. For AI models, SVR, ELM, and ANN are developed as single benchmark models. As a benchmark model for decomposition–reconstruction–ensemble models, four similar decomposition–reconstruction–ensemble frameworks with different basic prediction models are built.

## 4. Empirical Result

### 4.1. Research Data

In this paper, the weekly WTI and Brent crude oil spot price from the US Energy Information Administration (EIA) (http://www.eia.doe.gov/ (accessed on 11 August 2022)) were selected as sample data. The sampling period was from 1 January 2010 to 31 December 2021, and there are 627 observations in total. The training set accounts for 70% of the total sample size, which includes 418 observations, and the test set accounts for 30% of the total sample size, which includes 209 observations. The test data set is used to evaluate how well the proposed model performed compared to the benchmark models.

Table 1 displays these initial crude oil price series with their statistical measurements, which include the minima, maxima, means, and standard deviations. We find that the rejection of the null hypothesis of Gaussian distribution results from the Anderson and Darling test, which is confirmed by the time series data with nonzero skewness and positive excess kurtosis. Overall, the chosen observations are not stationary, and the model construction should consider necessary data preprocessing.

### 4.2. Experimental Result Analysis

**Figure 2:**
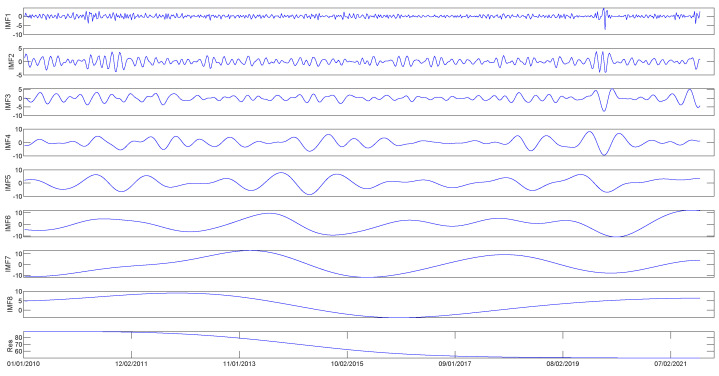
CEEMDAN decomposition results of the WTI crude oil prices (dollars per barrel).

**Figure 3:**
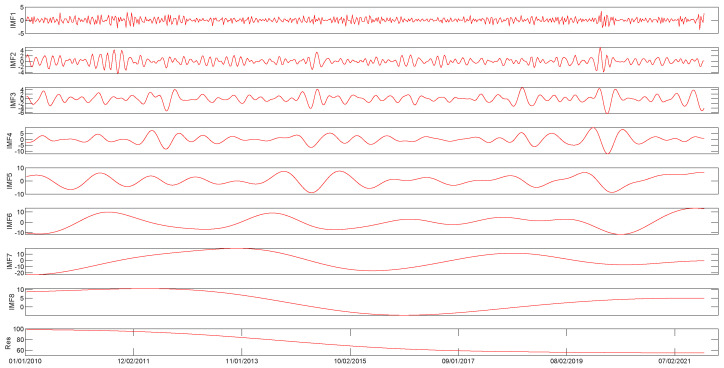
CEEMDAN decomposition results of the Brent crude oil prices (dollars per barrel).

First, the original time series of WTI and Brent crude oil prices are decomposed by CEEMDAN, as shown in Figure 2 and Figure 3. In particular, the price series of WTI and Brent crude oil are decomposed into 8 IMF components and one residual term. Each of the intrinsic mode functions can be categorized into high and low frequencies, with each component showcasing unique characteristics. The decomposition analysis reveals that the residue component exhibits noteworthy long-term trends, while sub-components 1 to 8 are stationary or nearly stationary, as illustrated in Figure 2 and Figure 3. However, the effectiveness of the decomposition process in improving crude oil price forecasting performance remains an open topic for further discussion in subsequent sections.

In the second step, component reconstruction is performed to reduce the computational time complexity. According to Table 2 and Table 3, different decomposed modes have different degrees of complexity, and the complexity traits of each decomposed mode show a downward trend with an increasing time scale. Subsequently, based on the recursive CEEMDAN algorithm, all components are recognized as high-complexity components, medium-complexity components, and low-complexity components. More concretely, IMFs and residual components are identified as low-complexity when they have smaller complexity traits than the original time series after the first decomposition. The IMFs with larger complexity traits than the original time series will be recognized as high-complexity components when they are recursively decomposed with only one step. Then, other IMFs are recognized as medium-complexity components. These components have larger complexity traits than the original time series, and they will be recursively decomposed with two or more steps. Table 2 and Table 3 report the test results of the complexity traits for each decomposed component of WTI and Brent crude oil prices, respectively.

Next, it is necessary to select a suitable method to predict different components. According to the complexity test results, nine components are reduced into six components after the reconstruction. In addition, the complexity traits of the decomposition components will change when the components change. Based on the reconstruction method, three kinds of components with different degrees of complexity, namely the high-complexity component, medium-complexity component, and low-complexity component, can be obtained. Then, the selection of suitable predictive methods driven by complexity traits is achieved through the trial-and-error approach. Table 4, Table 5, Table 6, Table 7, Table 8 and Table 9 presents the selection results for predicting different decomposed components of WTI and Brent crude oil prices with complexity traits.

Table 4, Table 5 and Table 6 show the performance value of different combination models such as X-SVR-SVR, SVR-X-SVR, and SVR-SVR-X. For example, in the X-SVR-SVR model (see Table 4), the second and third SVR methods indicate that the medium-complexity component and low-complexity component use the SVR model, while X will try four different methods (i.e., ES, SVR, ELM, ANN) to find a suitable model for the high-complexity component. To facilitate computational convenience, the ADD is temporarily employed as an ensemble method for investigating the correlation between the memorable component and the prediction method. Based on the aforementioned explanations, Table 4 presents the experimental findings regarding the high-complexity components.

For the parameter of the ES, a simple first-order ES with a smoothing constant is chosen. The smoothing constant is determined using the principle of the minimum root mean square error. For the parameters of the SVR model, the Gaussian RBF kernel function is adopted, and the grid search method is used to set the regularization and kernel parameters. For the ELM and ANN models, the number of nodes in the hidden layer is set to 30.

Table 4, Table 5 and Table 6 illustrate that an ANN is suitable for high-complexity component forecasting, while SVR is suitable for both medium-complexity and low-complexity component forecasting. The ANN-SVR-SVR has better prediction accuracy than other model combinations for WTI crude oil price forecasting. Table 7, Table 8 and Table 9 show the experimental results of the high-complexity component, medium-complexity component, and low-complexity component, respectively. Similarly, the SVR-SVR-SVR has better prediction performance than other model combinations for Brent crude oil price forecasting according to Table 7, Table 8 and Table 9.

## 5. Discussion

### 5.1. Prediction Performance Comparison

In this part, the proposed model, four single models (i.e., ES, SVR, ELM, ANN), and four decomposition–reconstruction–ensemble models (i.e., D-R-ES, D-R-SVR, D-R-ELM, D-R-ANN), which are considered benchmark models, are performed to predict the testing dataset of WTI and Brent crude oil prices. Here, “D” denotes the chosen decomposition method, and “R” denotes the proposed reconstruction rule of the component. The results are shown in Table 10, Table 11, Table 12 and Table 13. According to these results, the proposed model almost outperforms all of the considered benchmark models. The final form of the proposed model is simply the decomposition–reconstruction–ensemble model with the form of “D-R-SVR” for Brent crude oil price forecasting. Thus, the model with the form of “D-R-SVR” is not considered a target model in Table 13.

Furthermore, the decomposition–reconstruction–ensemble models make predictions better than the single models according to Table 10 and Table 11. In particular, for WTI crude oil price forecasting, the decomposition–reconstruction–ensemble models have average MAE, RMSE, and MAPE values of 1.0867, 1.6535, and 0.0345, respectively, while the single models have average MAE, RMSE, and MAPE values of 1.2334, 1.8382, and 0.0408, respectively. For the Brent crude oil price forecasting, the prediction accuracy values for the decomposition–reconstruction–ensemble models are 1.1463, 1.5782, and 0.0233, while those for the single models are 1.3429, 1.9266, and 0.0290. The main reason is that the decomposition–reconstruction–ensemble can minimize the complexity of crude oil data, which boosts its prediction performance compared to benchmark single models.

Comparing with the eight benchmark models, i.e., ES, SVR, ELM, ANN, D-R-ES, D-R-SVR, D-R-ELM, and D-R-ANN, the proposed model shows superior performance in crude oil price forecasting. In Table 10, the proposed model improves the prediction accuracy by 59.89%, 63.33%, and 61.82% on average compared to the benchmark single models and by 52.42%, 55.06%, and 53.10% on average compared to the benchmark decomposition–reconstruction–ensemble models. Then, Table 11 shows that the proposed model improves the accuracy of the Brent crude oil price forecasting by 62.01%, 65.88%, and 65.66% on average compared to the benchmark single models and by 52.96%, 51.17% and 51.47% on average compared with the benchmark decomposition–reconstruction–ensemble models. Therefore, the proposed recursive CEEMDAN decomposition–reconstruction–ensemble prediction method can effectively improve the prediction performance of WTI and Brent crude oil prices.

In addition, the DM test is used to compare the prediction performance of different models in the benchmark models in Table 12 and Table 13 to statistically prove the superiority of the proposed model for WTI and Brent crude oil price forecasting. These conclusions are statistically proven by data from the DM test, as indicated by the *p*-values (in brackets). First, at a significance level of 5%, the proposed model outperforms all benchmark models, which suggests that the proposed recursive CEEMDAN decomposition–reconstruction–ensemble prediction model is better than the listed benchmark models for WTI and Brent crude oil price forecasting. Second, when the decomposition–reconstruction–ensemble models in the benchmark models are tested as the target models in Table 12 and Table 13, only the D-R-SVR can be proven to be better than all single models with the significance level of 5%. Third, focusing on different decomposition–reconstruction–ensemble models in the benchmark models, although the D-R-SVR can be statistically demonstrated to be better than their D-R-based counterparts at the confidence level of 5%, it is essential to choose the appropriate prediction model for the reconstructed components with different degrees of complexity.

### 5.2. Further Discussion

**Figure 4:**
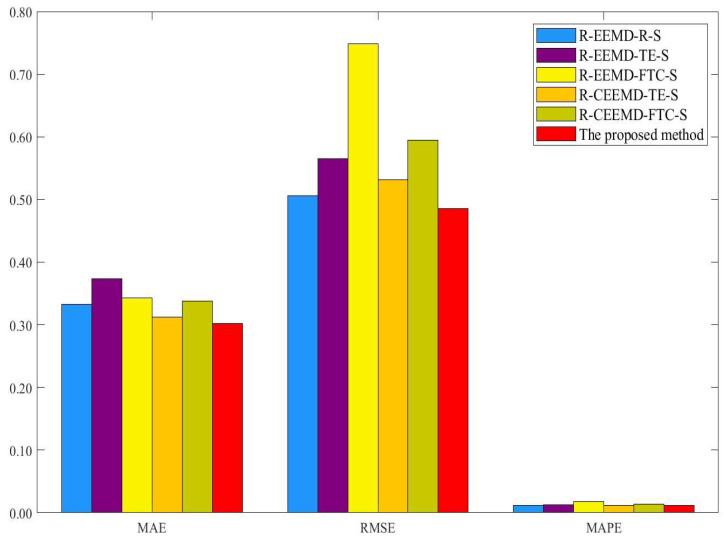
Performance comparison of different models for WTI crude oil price forecasting.

**Figure 5:**
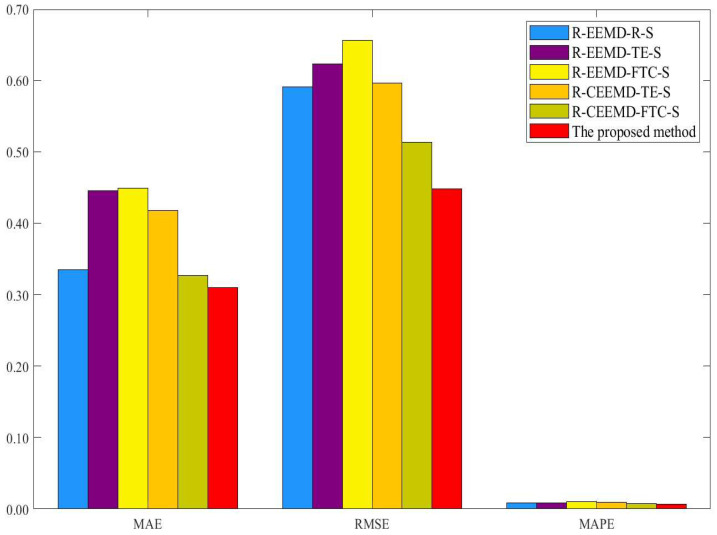
Performance comparison of different models for Brent crude oil price forecasting.

In this section, we perform the EEMD decomposition method and two different reconstruction rules to compare the prediction performance of the proposed model. The two rules are mode reconstruction based on the threshold setting of SE (see, e.g., Zhang et al. [39]) and fine-to-coarse (FTC) (see, e.g., Yu et al. [38] and Zhang et al. [39]). Different models are performed as the benchmark models, which are denoted in the form of R-D-R-SA, where “R-D” indicates different recursive decomposition methods to be compared, “R” indicates different reconstruction rules, and “SA” represents the selected predictive methods driven by the complexity traits and simple addition for the final ensemble. Table 14 and Table 15 and Figure 4 and Figure 5 show the results of different models. Similarly, the DM test is performed to evaluate the accuracy of different prediction models, and the corresponding results are presented in Table 16. According to Table 14, Table 15 and Table 16 and Figure 4 and Figure 5, the main findings are as follows.

First, as Table 14 and Table 15 show, no model can outperform other models under all indicators. Compared with the EEMD decomposition-based models, the proposed model for WTI crude oil price forecasting improves the prediction accuracy by 10.10%, 13.28%, and 11.35% on average, and the proposed model for Brent crude oil price forecasting improves the prediction accuracy by 17.27%, 21.0%, and 16.50% on average. One possible reason is that CEEMDAN minimizes the complexity of WTI and Brent crude oil price data. Thus, it can effectively filter out the meaningful components and significantly enhance the forecast accuracy.

Second, the proposed model is better than the benchmark models based on other reconstruction rules. In concrete, compared with the benchmark models with different reconstruction rules, the proposed model for WTI crude oil price forecasting improves the prediction accuracy by 3.51%, 6.75%, and 4.59% on average, and the proposed model for Brent crude oil price forecasting improves the prediction accuracy by 7.78%, 9.40%, and 7.05% on average. Table 16 also shows that the DM test at the 10% level of significance confirms the superiority of the suggested model. Thus, the WTI and Brent crude oil data can be better predicted using the proposed reconstruction approach based on the complexity trait.

Third, the proposed model has lower MAE, RMSE, and MAPE than other models based on the EEMD decomposition models and reconstruction rules from Figure 4 and Figure 5. For example, compared with different reconstruction methods in the benchmark models, the proposed model for WTI crude oil price forecasting improves the prediction accuracy by 10.89%, 16.03%, and 12.75% on average, and the proposed model for Brent crude oil price forecasting improves the prediction accuracy by 20.04%, 24.32%, and 18.84% on average. Thus, the proposed model improves the prediction performance in WTI and Brent crude oil price forecasting. Meanwhile, as shown in Table 16, when the proposed model is used as the target model, all p-values of the DM test fall below the threshold of 10%, so the proposed model has a significantly higher level of accuracy in its predictions than the benchmark models.

## 6. Conclusions and Future Directions

This paper proposes a new complexity-traits-driven recursively CEEMDAN decomposition–reconstruction–ensemble method for WTI and Brent crude oil price forecasting. All steps of component reconstruction for decomposed components, component prediction, and ensemble prediction are driven by the complexity traits, and the proposed method proves to be more effective than the benchmark models.

In the empirical analysis, the proposed recursive CEEMDAN decomposition– reconstruction–ensemble learning paradigm is significantly better than the most popular single models, different decomposition–reconstruction–ensemble models, and ensemble models based on the EEMD decomposition methods or different reconstruction rules. Based on the empirical experiments, four insightful conclusions can be summarized.

First, the prediction accuracy of WTI and Brent crude oil price data demonstrates that the proposed model outperforms all benchmark models. Specifically, compared with different benchmark models, the proposed model for WTI crude oil price forecasting improves the prediction accuracy by 56.16%, 59.19%, and 57.46% on average, and the proposed model for Brent crude oil price forecasting improves the prediction accuracy by 57.48%, 58.53%, and 58.56% on average. Therefore, the proposed model can be a useful tool to forecast WTI and Brent crude oil prices in the near future.

Second, CEEMDAN can achieve better prediction performance than the EEMD decom-position-based method. For example, compared with the EEMD decomposition-based models, on average, the proposed model improves the prediction accuracy by 10.10%, 13.28%, and 11.35% for WTI crude oil price forecasting and by 17.27%, 21.0%, and 16.50% for Brent crude oil price forecasting.

Third, the prediction performance of crude oil price data can be further improved by selecting appropriate prediction models for the reconstructed components with different degrees of complexity. For example, compared with the benchmark decomposition–reconstruction–ensemble models (i.e., D-R-KRR, D-R-ELM, D-R-SVR, and D-R-ANN), on average, the proposed model improves the prediction accuracy by 52.42%, 55.06%, and 53.10% for WTI crude oil price forecasting and by 52.96%, 51.17%, and 51.47% for Brent crude oil price forecasting. Therefore, it is essential to choose the appropriate prediction models according to the complexity traits.

Finally, compared with the existing reconstruction rules, the recursively decomposition-reconstruction method based on the complexity traits can reduce the modeling complexity well, which shows its usefulness and efficacy in WTI and Brent crude oil price forecasting. For example, on average, the proposed model improves the prediction accuracy by 10.89%, 16.03%, and 12.75% for WTI crude oil price forecasting and by 20.04%, 24.32%, and 18.84% for Brent crude oil price forecasting. Thus, mode reconstruction driven by complexity traits is effective.

In addition to the sample entropy used by our recursive CEEMDAN method, other time series features such as the frequency change rate and autocorrelation can be used. Future research extensions will focus on the following: (1) verifying more advanced decomposition methods under the proposed framework in this paper and (2) exploring more results in other research areas such as the stock market, power market, and other emerging markets using the proposed complexity-trait-driven reconstruction-ensemble learning paradigm.

## Figures and Tables

**Table 1:** Descriptive statistics of crude oil spot price data.

	Observations	Max	Min	Aver	Range	Std.dev.
	Skewness	Kurtosis	AD Test			
WTI	627	112.81	3.92	69.3636	108.89	22.3042
	0.0734	1.9038	10.4 ***			
Brent	627	126.62	14.24	75.6762	112.38	26.4016
	0.1842	1.8713	13.65 ***			

*** denotes rejections of the null hypothesis at the 1% level.

**Table 2:** Complexity test results of the IMF components for WTI crude oil.

IMFs	Sample Entropy	Complexity Traits
IMF1	1.7830	high-complexity
IMF2	1.0767	
IMF3	0.6369	
IMF4	0.4913	medium-complexity
IMF5	0.3749	
IMF6	0.1481	low-complexity
IMF7	0.0713	
IMF8	0.0262	
Res	0.0027	

**Table 3:** Complexity test results of the IMF components for Brent crude oil.

IMFs	Sample Entropy	Complexity Traits
IMF1	1.7466	high-complexity
IMF2	1.1038	
IMF3	0.6331	
IMF4	0.5276	medium-complexity
IMF5	0.3936	
IMF6	0.1420	low-complexity
IMF7	0.0591	
IMF8	0.0199	
Res	0.0019	

**Table 4:** Prediction performance comparison of a **high-complexity component** under different prediction methods for WTI crude oil price data.

MAE	ES	SVR	ELM	ANN
X-ES-ES	1.6191	0.8133	1.9097	**0.7849**
X-SVR-SVR	1.3695	0.3162	1.6804	**0.3021**
X-ELM-ELM	1.6175	0.8159	1.9106	**0.7875**
X-ANN-ANN	1.4411	0.5109	1.7242	**0.5010**
RMSE	ES	SVR	ELM	ANN
X-ES-ES	2.3884	1.1637	2.8503	**1.0986**
X-SVR-SVR	2.0536	0.6285	2.5845	**0.4857**
X-ELM-ELM	2.3882	1.1643	2.8512	**1.0093**
X-ANN-ANN	2.1183	0.8655	2.6417	**0.7459**
MAPE	ES	SVR	ELM	ANN
X-ES-ES	0.0470	0.0239	0.0583	**0.0204**
X-SVR-SVR	0.0426	0.0149	0.0544	**0.0116**
X-ELM-ELM	0.0470	0.0239	0.0583	**0.0204**
X-ANN-ANN	0.0450	0.0210	0.0562	**0.0178**

**Table 5:** Prediction performance comparison of a **medium-complexity component** under different prediction methods for WTI crude oil price data.

MAE	ES	SVR	ELM	ANN
ES-X-ES	1.6191	**1.4027**	1.6186	1.4060
SVR-X-SVR	0.7643	**0.3162**	0.7647	0.3649
ELM-X-ELM	1.9101	1.7173	1.9106	**1.7136**
ANN-X-ANN	0.8195	**0.4422**	0.8199	0.5010
RMSE	ES	SVR	ELM	ANN
ES-X-ES	2.3884	**2.0670**	2.3883	2.0730
SVR-X-SVR	1.0727	**0.6285**	1.0729	0.6910
ELM-X-ELM	2.8510	**2.5929**	2.8512	2.5999
ANN-X-ANN	1.1255	**0.6794**	1.1257	0.7459
MAPE	ES	SVR	ELM	ANN
ES-X-ES	0.0470	**0.0430**	0.0470	0.0433
SVR-X-SVR	0.0231	**0.0149**	0.0231	0.0164
ELM-X-ELM	0.0583	**0.0550**	0.0583	**0.0550**
ANN-X-ANN	0.0223	**0.0162**	0.0223	0.0178

**Table 6:** Prediction performance comparison of a **low-complexity component** under different prediction methods for WTI crude oil price data.

MAE	ES	SVR	ELM	ANN
ES-ES-X	1.6191	1.6247	**1.6170**	1.6973
SVR-SVR-X	0.4036	**0.3162**	0.4050	0.4554
ELM-ELM-X	1.9084	**1.9032**	1.9106	1.9668
ANN-ANN-X	0.4123	**0.3561**	0.4143	0.5010
RMSE	ES	SVR	ELM	ANN
ES-ES-X	2.3884	**2.3445**	2.3882	2.3963
SVR-SVR-X	0.6679	**0.6285**	0.6684	0.8033
ELM-ELM-X	2.8504	**2.8165**	2.8512	2.8629
ANN-ANN-X	0.5871	**0.5563**	0.5878	0.7459
MAPE	ES	SVR	ELM	ANN
ES-ES-X	0.0470	0.0471	**0.0469**	0.0494
SVR-SVR-X	0.0163	**0.0149**	0.0163	0.0194
ELM-ELM-X	0.0583	**0.0581**	0.0583	0.0603
ANN-ANN-X	0.0141	**0.0132**	0.0141	0.0178

**Table 7:** Prediction performance comparison of a **high-complexity component** under different prediction methods for Brent crude oil price data.

MAE	ES	SVR	ELM	ANN
X-ES-ES	1.6801	**0.8727**	2.0259	0.8862
X-SVR-SVR	1.3609	**0.3096**	1.7325	0.3148
X-ELM-ELM	1.6803	**0.8732**	2.0269	0.8872
X-ANN-ANN	1.4301	**0.5547**	1.7848	0.5687
RMSE	ES	SVR	ELM	ANN
X-ES-ES	2.3661	**1.2018**	2.7537	1.2179
X-SVR-SVR	1.8842	**0.4478**	2.3509	0.4611
X-ELM-ELM	2.3661	**1.2018**	2.7542	1.2180
X-ANN-ANN	1.9614	**0.7250**	2.4200	0.7447
MAPE	ES	SVR	ELM	ANN
X-ES-ES	0.0332	**0.0180**	0.0401	0.0183
X-SVR-SVR	0.0269	**0.0071**	0.0341	0.0072
X-ELM-ELM	0.0332	**0.0180**	0.0401	0.0183
X-ANN-ANN	0.0287	**0.0127**	0.0349	0.0129

**Table 8:** Prediction performance comparison of a **medium-complexity component** under different prediction methods for Brent crude oil price data.

MAE	ES	SVR	ELM	ANN
ES-X-ES	1.6801	**1.3943**	1.6815	1.4119
SVR-X-SVR	0.7827	**0.3096**	0.7840	0.3126
ELM-X-ELM	2.0282	**1.7682**	2.0269	1.7719
ANN-X-ANN	0.9064	**0.4804**	0.9052	0.5687
RMSE	ES	SVR	ELM	ANN
ES-X-ES	2.3661	**1.9098**	2.3662	1.9345
SVR-X-SVR	1.0163	**0.4478**	1.0169	0.4989
ELM-X-ELM	2.7549	**2.3742**	2.7542	2.3938
ANN-X-ANN	1.1946	**0.6384**	1.1943	0.7447
MAPE	ES	SVR	ELM	ANN
ES-X-ES	0.0332	**0.0279**	0.0333	0.0284
SVR-X-SVR	0.0159	**0.0071**	0.0159	0.0076
ELM-X-ELM	0.0401	0.0348	0.0401	**0.0347**
ANN-X-ANN	0.0185	**0.0111**	0.0185	0.0129

**Table 9:** Prediction performance comparison of a **low-complexity component** under different prediction methods for Brent crude oil price data.

MAE	ES	SVR	ELM	ANN
ES-ES-X	1.6801	**1.6119**	1.6815	1.7146
SVR-SVR-X	0.3768	**0.3096**	0.3780	0.4635
ELM-ELM-X	2.0246	**1.9625**	2.0269	2.0606
ANN-ANN-X	0.4521	**0.3216**	0.4544	0.5687
RMSE	ES	SVR	ELM	ANN
ES-ES-X	2.3661	**2.2772**	2.3662	2.3468
SVR-SVR-X	0.5445	**0.4478**	0.5451	0.6216
ELM-ELM-X	2.7533	**2.6762**	2.7542	2.7416
ANN-ANN-X	0.6444	**0.5183**	0.6453	0.7447
MAPE	ES	SVR	ELM	ANN
ES-ES-X	0.0332	**0.0317**	0.0333	0.0337
SVR-SVR-X	0.0091	**0.0071**	0.0091	0.0108
ELM-ELM-X	0.0401	**0.0386**	0.0401	0.0405
ANN-ANN-X	0.0109	**0.0079**	0.0109	0.0129

**Table 10:** Performance comparison of WTI crude oil spot price data.

Model	MAE	RMSE	MAPE
ES	1.9048	2.5800	0.0583
SVR	0.3858	0.8642	0.0201
ELM	1.9102	2.8511	0.0583
ANN	0.5857	0.8727	0.0203
D-R-ES	1.6191	2.3884	0.0470
D-R-SVR	0.3162	0.6285	0.0149
D-R-ELM	1.7635	2.6664	0.0520
D-R-ANN	0.5010	0.7459	0.0178
Proposed Model	**0.3021**	**0.4857**	**0.0116**

**Table 11:** Performance comparison of Brent crude oil spot price data.

Model	MAE	RMSE	MAPE
ES	2.0208	2.7528	0.0400
SVR	0.3811	0.6554	0.0104
ELM	2.0270	2.7542	0.0401
ANN	0.7461	1.1956	0.0198
D-R-ES	1.6801	2.3661	0.0332
D-R-SVR	**0.3096**	**0.4478**	**0.0071**
D-R-ELM	1.8305	1.4877	0.0332
D-R-ANN	0.5687	0.7447	0.0129
Proposed Model	**0.3096**	**0.4478**	**0.0071**

**Table 12:** Performance comparison of WTI crude oil spot price data with DM test.

	D-R-ES	D-R-SVR	D-R-ELM	D-R-ANN	ES	SVR
	ELM	ANN				
Proposed Model	−5.0627 (***)	−2.2336 (**)	−4.6018 (***)	−4.7393 (***)	−4.5974 (***)	−1.8938 (**)
	−4.6011 (***)	−4.4155 (***)				
D-R-ES		5.1956	−2.5090 (***)	4.9168	−2.5018 (***)	5.2711
	−2.5084 (***)	4.8856				
D-R-SVR			−4.7382 (***)	−1.7617 (**)	−4.7335 (***)	−1.7749 (**)
	−4.7374 (***)	−4.2466 (***)				
D-R-ELM				4.5240	0.9932	4.9374
	−1.2236	4.5142				
D-R-ANN					−4.5195 (***)	−0.7064
	−4.5233 (***)	−3.4376 (***)				
ES						4.9323
	−0.9629	4.5096				
SVR						
	−4.9365 (***)	−0.0656				
ELM						
		4.5134				

*** and ** denote 1% significant level and 5% significant level.

**Table 13:** Performance comparison of Brent crude oil spot price data with DM test.

	D-R-ES	D-R-ELM	D-R-ANN	ES	SVR
	ELM	ANN			
Proposed Model	−4.7937 (***)	−4.8754 (***)	−5.3732 (***)	−4.8701 (***)	−2.0571 (**)
	−4.8761 (***)	−3.9619 (***)			
D-R-ES		−3.1369 (***)	4.5017	−3.1250 (***)	4.6181
	−3.1378 (***)	3.7919			
D-R-ELM			4.6596	0.9593	4.7483
	−0.2304	4.1415			
D-R-ANN				−4.6543 (***)	−1.8099
	−4.6603 (***)	−3.5135 (***)			
ES					4.7429
	−0.9702	4.1362			
SVR					
	−4.7490 (***)	−4.6727 (***)			
ELM					
		4.1432			

*** and ** denote 1% significant level and 5% significant level.

**Table 14:** Performance comparison of WTI crude oil spot price data with EEMD decomposition method and different reconstruction rules.

Model	MAE	RMSE	MAPE
R-EEMD-R-SA	0.3332	0.5057	0.0121
R-EEMD-TE-SA	0.3738	0.5651	0.0124
R-EEMD-FTC-SA	0.3428	0.7486	0.0178
R-CEEMD-TE-SA	0.3128	0.5319	0.0122
R-CEEMD-FTC-SA	0.3380	0.5946	0.0134
Proposed Model	**0.3021**	**0.4857**	**0.0116**

**Table 15:** Performance comparison of Brent crude oil spot price data with EEMD decomposition method and different reconstruction rules.

Model	MAE	RMSE	MAPE
R-EEMD-R-SA	0.3350	0.5906	0.0087
R-EEMD-TE-SA	0.4450	0.6231	0.0088
R-EEMD-FTC-SA	0.4492	0.6555	0.0099
R-CEEMD-TE-SA	0.4175	0.5958	0.0097
R-CEEMD-FTC-SA	0.3269	0.5134	0.0072
Proposed Model	**0.3096**	**0.4478**	**0.0071**

**Table 16:** DM test results across different models for WTI and Brent crude oil price forecasting.

	R-EEMD-R-S	R-EEMD-TE-S	R-EEMD-FTC-S	R-CEEMD-TE-S	R-CEEMD-FTC-S
Proposed Model (WTI)	−2.1569 (**)	−3.1099 (***)	−1.3059 (*)	−3.8245 (***)	−1.5388 (*)
Proposed Model (Brent)	−2.4887 (***)	−34.0993 (***)	−3.2081 (***)	−1.9624 (**)	−1.8207 (**)

***, ** and * denote 1% significant level, 5% significant level and 10% significant level.

## Data Availability

http://www.eia.doe.gov/ (accessed on 11 August 2022).
